# The effects of plant cysteine proteinases on the nematode cuticle

**DOI:** 10.1186/s13071-021-04800-8

**Published:** 2021-06-05

**Authors:** Victor S. Njom, Tim Winks, Oumu Diallo, Ann Lowe, Jerzy Behnke, Mark J. Dickman, Ian Duce, Iain Johnstone, David J. Buttle

**Affiliations:** 1 Department of Infection, Immunity and Cardiovascular Disease, The University of Sheffield Medical School, Beech Hill Road, Sheffield, S10 2RX UK; 2 Department of Applied Biology and Biotechnology, Enugu State University of Science and Technology, Enugu, 1660 PMB Nigeria; 3 Department of Biosciences and Chemistry, Sheffield Hallam University, Sheffield, S1 1WB UK; 4 School of Life Sciences, University of Nottingham, University Park, Nottingham, NG7 2RD UK; 5 Department of Chemical and Biological Engineering, ChELSI Institute, The University of Sheffield, Sheffield, S1 3JD UK; 6 Department of Life Sciences and Biomolecular Sciences, University of Glasgow, Glasgow, UK

**Keywords:** *C. elegans*, *H. bakeri*, Papain, Papaya latex, Cuticle, Anthelmintic, Proteomics, Imaging, Immunohistochemistry

## Abstract

**Background:**

Plant-derived cysteine proteinases of the papain family (CPs) attack nematodes by digesting the cuticle, leading to rupture and death of the worm. The nematode cuticle is composed of collagens and cuticlins, but the specific molecular target(s) for the proteinases have yet to be identified.

**Methods:**

This study followed the course of nematode cuticle disruption using immunohistochemistry, scanning electron microscopy and proteomics, using a free-living nematode, *Caenorhabditis elegans* and the murine GI nematode *Heligmosomoides bakeri* (*H. polygyrus*) as target organisms.

**Results:**

Immunohistochemistry indicated that DPY-7 collagen is a target for CPs on the cuticle of *C. elegans*. The time course of loss of DPY-7 from the cuticle allowed us to use it to visualise the process of cuticle disruption. There was a marked difference in the time course of damage to the cuticles of the two species of nematode, with *H. bakeri* being more rapidly hydrolysed*.* In general, the CPs’ mode of attack on the nematode cuticle was by degrading the structural proteins, leading to loss of integrity of the cuticle, and finally death of the nematode. Proteomic analysis failed conclusively to identify structural targets for CPs, but preliminary data suggested that COL-87 and CUT-19 may be important targets for the CPs, the digestion of which may contribute to cuticle disruption and death of the worm. Cuticle globin was also identified as a cuticular target. The presence of more than one target protein may slow the development of resistance against this new class of anthelmintic.

**Conclusions:**

Scanning electron microscopy and immunohistochemistry allowed the process of disruption of the cuticle to be followed with time. Cuticle collagens and cuticlins are molecular targets for plant cysteine proteinases. However, the presence of tyrosine cross-links in nematode cuticle proteins seriously impeded protein identification by proteomic analyses. Multiple cuticle targets exist, probably making resistance to this new anthelmintic slow to develop.

**Graphic Abstract:**

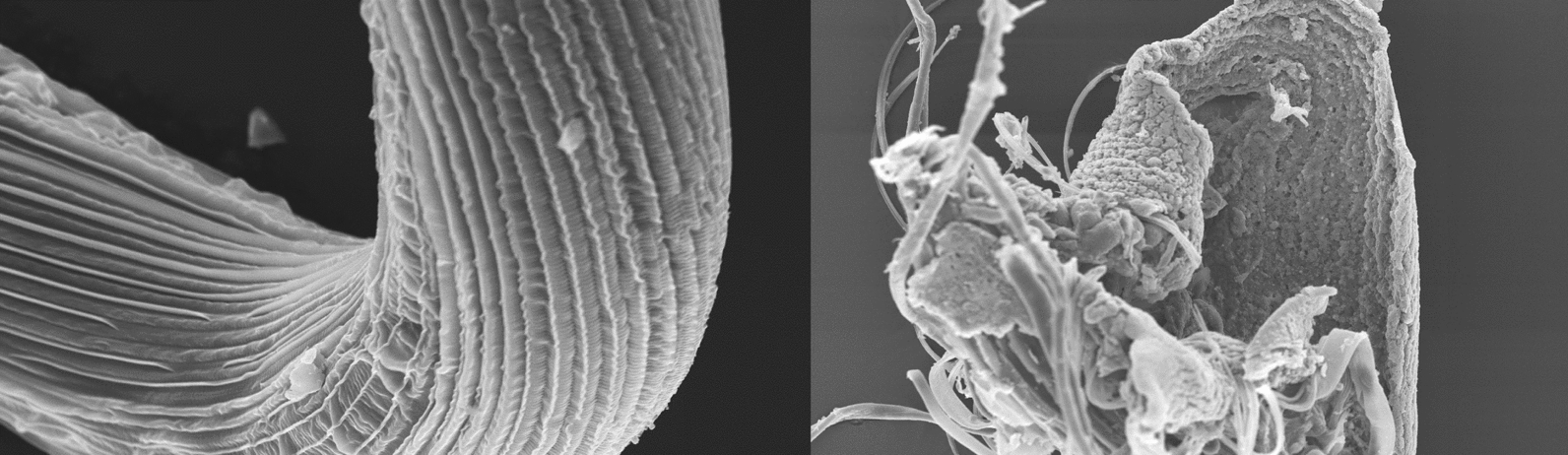

**Supplementary Information:**

The online version contains supplementary material available at 10.1186/s13071-021-04800-8.

## Background

Parasitic nematodes cause enormous public health, agricultural and economic problems worldwide, as pathogens of humans [1, 2], livestock [3] and crops [4]. In humans, treatment of gastrointestinal (GI) nematode/soil-transmitted helminth (STH) infections is usually with one or a combination of two or all three common classes of synthetic anthelmintics: benzimidazoles, nicotinic acetylcholine agonists and macrocyclic lactones [5], whose modes of action range from binding to microtubules and blockage of metabolic pathways to effects on neuromuscular transmission [6]. The intensive use of drugs and the dependence of treatment of nematode infection on only a few drugs with similar modes of action have put pressure on the drug candidates with resulting loss of potency due to development of resistance by target nematodes [7–9]. Nematode resistance to anthelmintics is a crisis in certain livestock industries, particularly in small ruminant animals, where triple-resistant nematodes have been reported [10]. Though the greatest problem is in treatment of ruminants, there are signs that resistance is also developing in human populations [7, 8, 11, 12].

Nematodes are protected from their environment by their cuticle, which also confers shape and integrity to the worms. The cuticle is made of two important structural protein types, collagens and cuticlins, encoded by about 160 and at least 8 genes, respectively, in *C. elegans*. These structural proteins are strengthened by the presence of disulphide and tyrosine-based cross-links [13–15]. The parasitic nematode species that inhabit the gastrointestinal tract produce proteinase inhibitors of serine proteinases and pepsin, and hence are able to avoid being digested and can survive in the gut lumen [16–18]. However, in the GI tract they are not exposed to high concentrations of cysteine proteinases (CPs) and therefore do not experience selective pressure to develop inhibitors to this class of proteinase in order to survive in the gut. Their protective cuticle may therefore be sensitive to digestion by this class of proteinases. Tropical countries have relied to some extent on plant extracts for the treatment of nematode infections [19], including extracts that contain CPs [20–22].

Many plant CPs are in the papain family (subfamily C1A in the phylogenetic classification in the *MEROPS* database—http://merops.sanger.ac.uk/) [23]. They attack the nematode cuticle, weakening its structure sufficiently to allow the internal high hydrostatic pressure in the pseudocoelomic cavity to rupture the cuticle, resulting in evisceration and death of the worm. This mode of action appears to be the same both in vitro and in vivo [24–27]. Free-living and plant parasitic nematodes undergo the same fate as animal GI nematodes [28–30].

To accomplish growth, the cuticle is shed five times during the life of a nematode in a process known as moulting or ecdysis [15]. This involves the digestion of the old cuticle by cysteine and metalloproteinases [15, 31]. It is possible that the anthelmintic action of plant CPs may therefore mimic the process of removal of the old unwanted cuticle during moulting.

For CPs to be accepted as an anthelmintic for livestock or for human use, we need to understand more about the mode of action, safety and toxicity. We have therefore investigated cuticle disruption by CPs of a well-annotated free-living nematode, *C. elegans*, using an immunohistochemical approach, then described the time-dependent process of cuticle digestion of *C. elegans* and a murine GI nematode, *Heligmosomoides bakeri*, using scanning electron microscopy and immunohistochemistry. We also undertook a proteomic approach in an attempt to identify the molecular targets for CPs. The presence of multiple targets for CPs in the cuticle is highly likely to decrease the chance of future resistance developing against the drug.

## Methods

### *C. elegans* culture

The *C. elegans* genome contains two cystatins, the functions of which include the inhibition of papain-like CPs [29]. The following *C. elegans* strains were used in this study: Bristol N2 wild type (WT), the cystatin gene null mutant RB1207 *cpi*-2(*ok1256*) [29] and cuticle collagen gene mutant dpy-7*(qm63)* [32]. We used a slight modification of the protocol described by Stiernagle in www.wormbook.org [13]. The *C. elegans* strains were cultured on plates of nematode growth medium (NGM) agar spread with an *Escherichia coli* (OP50) lawn. Worms from each plate were washed with approximately 10 ml of ice-cold M9 buffer into 50 ml sterile centrifuge tubes. The worms were settled on ice for 15 min, and the supernatant containing food bacteria was removed with a Pasteur pipette, leaving the worm suspension. Twenty millilitres of 60% (w/v) sucrose was added to the tube and mixed by inversion then centrifuged at 121×*g* for 2 min. Ten millilitres of this suspension containing the worms was aspirated into a new tube and washed twice with ice-cold M9 by centrifuging at 121×*g* for 2 min. The agar debris and bacterial sediments at the bottom of the tube were discarded. Worms were aliquoted in volumes of 1 ml (~ 4500 worms) and stored at −20 °C until use. To obtain a synchronised population, we used a modification of the protocol described by Stiernagle in www.wormbook.org [13], and adult worms were washed off the plates with K medium (prepared as 53 mM NaCl, 32 mM KCl). The worm suspension was passed through a 5 µm microplate sieve to remove any L1 and L2 larval stages. The resulting suspension was centrifuged at 755×*g* for 30 min. The supernatant was removed from the tube without disturbing the worms and replaced with egg isolation bleach (1% sodium hypochlorite and 0.5% KOH). The tubes were shaken for 7 min to disrupt the worms and release their eggs, then the tube was centrifuged for 3 min at 755×*g*. The supernatant was replaced with fresh K medium, and the process was repeated three times to remove any trace of the bleach solution. The tube was shaken on a rotary shaker overnight to allow L1 to hatch. The contents of the tube were then allowed to settle, and the supernatant was removed, leaving 2 ml in the 50 ml tube, which was transferred to several NGM agar plates with the aid of a pipette and incubated at 15 °C for 24, 39, 55, 74 or 95 h to obtain L2, L2–L3, L3–L4, L4 and adult worms, respectively. All experiments on *C. elegans* described in this paper were undertaken using worms harvested after 95 h.

### *Heligmosomoides bakeri *culture

We used the method described by Behnke and Harris [33]. Briefly, oral gavage with a blunt-ended needle was used to infect 7-week-old BKW mice with L3 of *H. bakeri* (Home Office Licence 40/3138) [34]. The mice were housed and maintained at the University of Nottingham, BioSupport Unit. Mice were provided with water and food ad libitum. At least 2 weeks post-infection, the mice were sacrificed by asphyxiation with CO_2_ and dissected. The intestine was carefully removed and placed inside a 15 cm-diameter Petri dish containing pre-warmed (37 °C) Hanks’ balanced salt solution (HBSS). To quicken the emergence of the adult worms from the mouse intestinal lumen, the intestine was carefully slit open longitudinally and incubated in HBSS or suspended in gauze in HBSS in a 50 ml beaker kept in a 37 ºC water bath. Worms collecting in the bottom of the beaker were tipped into a Petri dish, and with the aid of a stereomicroscope, adult worms that had migrated out of the gut lumen were pipetted or picked up with the aid of fine forceps and transferred into another Petri dish containing HBSS. Worms were later separated into males and females, and aliquots were stored in 2 ml mini-fuge tubes at −20 °C.

### Preparation of worm cuticles

We used a modification of the method described by Cox et al. [35]. An aliquot of either *C. elegans* strains or *H. bakeri* (containing ~ 4500 *C. elegans* or ~ 120 *H bakeri* adult worms) in a 1.5 ml mini-fuge tube was thawed, and 1 ml of H_2_O was added and vortexed to mix. The mini-fuge tube was centrifuged, and the water was decanted. Following phosphate-buffered saline (PBS) washes, 1 ml of 1% (w/v) sodium dodecyl sulphate (SDS) in 0.125 M Tris–HCl (pH 6.8) was added to the pellet, boiled for 5 min, incubated at ambient temperature for 1 h and centrifuged at 121×*g* for 5 min, and the supernatant was taken off. The procedure was repeated for *H. bakeri* but not for *C. elegans*, because *C. elegans* fragmented and lost their intact morphology. After the SDS wash, the worm pellet was washed again in PBS and centrifuged at 121×*g*, and the last supernatant was taken off. The prepared worm cuticles were finally washed in H_2_O and stored in PBS at −20 °C until they were used. β-Mercaptoethanol [35] was excluded in this procedure because it fragmented the cuticles, leading to loss of their intact cylindrical form.

### Preparation of CPs

The two preparations of CPs used in this study were purified papain from papaya latex, purchased from Sigma-Aldrich UK (product no. P3125, 2× crystallised aqueous suspension) and papaya latex supernatant (PLS), prepared as described previously [26]. PLS contains a mixture of four papaya CPs: chymopapain, glycyl endopeptidase, caricain and papain (in order of abundance) [36]. On the day of use, the enzyme preparations were titrated for the molar concentration of active enzyme, using the irreversible CP inactivator L-*trans*-epoxysuccinyl-leucylamido(4-guanidino)butane (E64) (Sigma-Aldrich product no. E3132) [37, 38]. The active enzyme concentration was diluted with water to give a 4 µM stock.

### Immunohistochemistry

The *C. elegans* collagen gene *dpy-7* knockout affects body shape (dumpy) [39, 40]. The DPY-7 cuticle collagen is predicted to have a carboxyl-terminal domain of 40 residues that is not shared with other *C*. *elegans* cuticle collagens [39]. The DPY-7-5a monoclonal antibody recognises specifically this “C”-terminal region of DPY-7 [39]. Using this antibody, we predicted that the presence or absence of a signal detection from cuticles with or without digestion by CP will indicate whether DPY-7 collagen is degraded or not by the CP. Additionally, DPY-7 immunohistochemistry can be used to monitor changes in cuticle structure during digestion of the cuticle components by a CP. For these experiments, we used wild-type (WT) *C. elegans*, and *dpy-7* null strain MQ375. We used two slightly different methodologies; the first used a mini-fuge tube, and the second was performed in 24-well plates. We used the mini-fuge tube method because we suspected disturbance and possible breaking of worms during centrifugation, whereas in well plates, there was minimal or no disturbance of worms.

In the tube experiments, aliquots of washed WT or mutant *dpy-7(qm63) C. elegans* were thawed and rinsed with water by centrifugation at 121×*g* for 2 min. The worms were partially reduced and made permeable with 1% dithiothreitol (DTT) or not [41], and were then incubated with 1 µM papain or PLS, or papain or PLS + 1 mM E64, at time points of 5, 10, 15 and 30 min at 37 °C. Enzyme activity was then stopped with 1 mM E64. The worms were washed with Tris-buffered saline (pH 7.0) with Tween 20 (TBST) by centrifugation at 121×*g* for 4 min. The washing was repeated three more times to remove any trace of CP, and non-specific binding sites were blocked for 4 h with 750 µl of 5% skimmed milk in TBST. The worms were probed with 1 ml of a 1:200 dilution of DPY-7 antibody for 4 h or overnight, followed by 1 ml of a 1:500 dilution of goat anti-mouse IgG secondary antibody Alexa Fluor 488 conjugate (Thermo Fisher Scientific, UK) in the dark for 2 h, and from there, samples were protected from light by wrapping in aluminium foil. The worm samples were centrifuged at 121×*g* for 2 min. The washing was repeated twice. After washing, 10 µl of worm suspension was pipetted onto a grease-free slide and mixed with mounting medium for fluorescence analysis (Vectashield H-1200) and protected with a coverslip.

In the 24-well plate method, all the conditions were the same as in the tube method except that the worms were not washed by centrifugation, but manually by pipetting the reagent with minimal disturbance to the worms which were not made permeable with 1% DTT. It is important to note that in all cases, the CP activity was totally eliminated by washing the samples in 1 mM E64, followed by three washes in TBST for 4 min before application of antibody, eliminating the possibility of hydrolysis of the antibody by CP [42]. The worms were imaged with a DMI4000 B (Leica) inverted widefield fluorescence microscope, and the images were stored electronically.

### Scanning electron microscopy (SEM)

Whole nematodes were used for this experiment. Approximately 30 *C. elegans* or 10 *H. bakeri* were added into each of four 1.5 ml mini-fuge tubes. The worms were incubated with 1 µM (final concentration) of CP or CP + E64 at a temperature of 37 °C for 10, 15 and 30 min. At each time point, activity of CPs was stopped with 50 µl of 1 mM E64. The samples were then diluted with PBS and centrifuged at 121×*g* for 2 min, and the supernatant was removed. This washing step was repeated three times to remove any trace of CP. The samples were fixed in 2.5% glutaraldehyde in 0.1 M phosphate buffer (pH 6.8) for 1 h, before being washed for 20 min three times in PBS then fixed and stained with 1% osmium tetroxide in 0.1 M phosphate buffer pH 6.8 for 1 h at ambient temperature. The samples were washed three times in water and dehydrated by sequentially placing in 30%, 50%, 70%, 90% and 100% ethanol. The specimens were then dried using a Polaron E3000 critical point dryer. The dried samples were mounted onto aluminium stubs using carbon discs. The stubs were gold sputter-coated (approximately 10 nm thick) using a Polaron E5100 SEM coating unit. All specimens were viewed and photographed using a JEOL JSM-840 scanning electron microscope at 23 kV, and the images were stored electronically.

### Digestion of worm cuticles with CPs for proteomic analyses

An aliquot (~ 4500 *C. elegans* or 120 *H. bakeri*) of either prepared worm cuticles or whole worms was incubated in 1 µM papain (final active concentration) or PLS (both activated with 4 mM l-cysteine), or papain or PLS + 1 mM E64 as the control, at 37 °C for 10, 15 and 30 min. Twenty-five microlitres of the supernatant was collected at each time point and mixed with 20 µl of 1 mM E64 to stop further CP activity.

### Sodium dodecyl sulphate–polyacrylamide gel electrophoresis (SDS-PAGE) of CP digested worm supernatant

The supernatant was mixed at a ratio of 1:1 with 2× sample buffer [4% SDS, 20% glycerol, 10% DTT, 0.004% bromophenol blue and 0.125 M Tris–HCl pH 6.5] and boiled for 5 min. Twenty microlitres of the boiled sample was loaded onto a 12% or 15% polyacrylamide 12-well precast Mini Protean gel (Bio-Rad). Following electrophoresis at 120 V, the gel was removed and fixed for 30 min in 5 ml of 7% (v/v) glacial acetic acid in 40% (v/v) methanol. Later, the gel was stained with 150 ml of 0.25% (w/v) colloidal Coomassie brilliant blue G concentrate in 50% methanol, 10% acetic acid and 40% water for at least 4 h. After staining, the gel was rinsed with 10% acetic acid in 40% methanol for 1 min. Rinsing was repeated, and the gel was de-stained overnight in 25% methanol on a shaker at ambient temperature. The next day the gel was washed, scanned using a Bio-Rad gel imager (Gel Doc XR^+^ System) and recovered and fixed in 1% formic acid.

### Peptide extraction and mass spectroscopy (LC/MS/MS)

In-gel tryptic digestion was a slight modification of the method described previously [43, 44] (see attached Additional file 1).

## Results

### Effects of CPs on the DPY-7 cuticle collagen of C. elegans

**Fig. 1:**
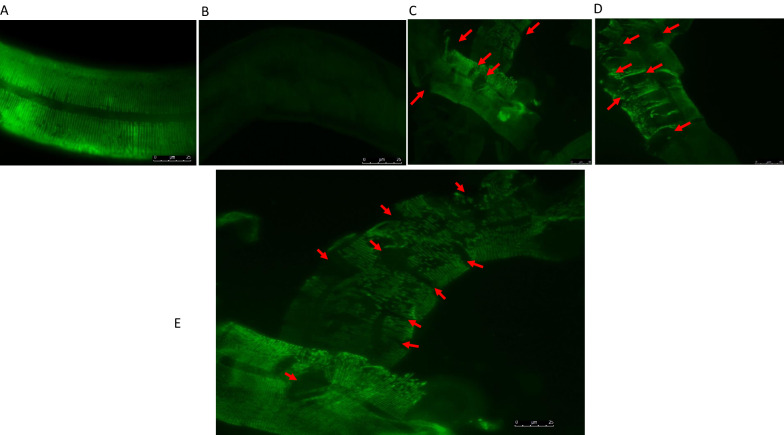
Immunohistochemical investigation of the activities of papain on prepared cuticles of WT (Bristol N2) *C. elegans*. The worm cuticles were partially reduced with 1% DTT to increase permeability using the tube method then probed with the DPY-7 antibody. **b** Mutant *dpy-7(qm63) C. elegans* used as the negative control. **c** and **d** Disruption of the WT cuticle after 5 min of incubation in 1 µM papain. **a** A worm incubated in 1 µM papain inactivated with E64 prior to incubation. **e** A magnified image showing the regular pattern of disruption (arrowed red) and progressive disappearance of DPY-7 at 5 min of incubation with 1 µM papain, indicating the sequence of events leading to the collapse of the cuticle structure. Bar = 25 µm

**Fig. 2:**
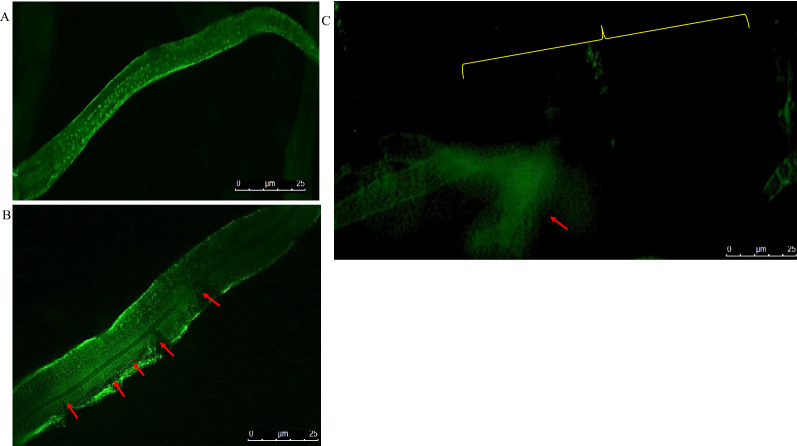
Immunolocalisation of DPY-7 in WT *C. elegans* (Bristol N2) cuticles incubated with papain or papain + E64 in a 24-well plate. Whole intact worms were incubated with 1 µM papain or papain + E64 without prior reduction in 1% DTT. **a** A worm incubated with papain + E64 for 30 min. **b** Disruption of the *C. elegans* (Bristol N2 WT) cuticle (arrowed red) after 5 min incubation. **c** Total disappearance of the collagen stripes and loss of immunoreactivity (yellow brackets) after incubation for 30 min. Bar = 25 µm

Figure 1 illustrates the immunochemical staining of the cuticles of *C. elegans* after incubation with 1 µM papain. The DPY-7 collagen locates to parallel circumferential thread-like bands within the cuticle [32]. When WT *C. elegans* cuticles were incubated for 5 min in 1 µM papain plus the CP inhibitor E64, a WT pattern of localisation of DPY-7 was observed (Fig. 1a) where the DPY-7 circumferential thread-like bands are intact and appear the same as for untreated specimens [32]. Staining of the *C. elegans* mutant strain *dpy-7(qm63)* was performed as a negative control; this strain lacks the DPY-7 collagen and hence has no staining for DPY-7 (Fig. 1b). When WT *C. elegans* cuticles were incubated with 1 µM papain for 5 min, frequent areas of structural disruption of the circumferential band structures were observed (arrowed red in Fig. 1c–e). The areas of major disruption appeared to be relatively regularly spaced (between 4 and 6 µm), although many parts of the cuticles had also lost some circumferential bands. Both the alae and the entire cuticle components totally disappeared after 10 min of incubation. This suggests that DPY-7 is a target protein for papain. Figure 2 shows representative images following the immunochemical staining of the cuticles of WT *C. elegans* after worms were incubated with 1 µM papain or papain + E64 in a 24-well plate without prior reduction in 1% DTT. The advantage of the plate method was that the worms were not disturbed by centrifugation, which allowed us to monitor progressively the activity of CP on the worms. The images presented here were of treated worms lying at the bottom of the wells, which were imaged without transferring to microscope slides. Worms incubated in papain for 5 min (Fig. 2b) were disrupted in the same regular pattern (red arrows) as was seen in worms prepared using the tube method (Fig. 1). This is in contrast with worms incubated in 1 µM papain + E64 (Fig. 2a). In Fig. 2c, after 30 min in 1 µM papain, the DPY-7 staining had mostly disappeared, and what was left had very little resolution (arrowed red). Some of the DPY-7 fluorescence remained until the cuticle was almost totally disrupted, indicating that this collagen species, or other proteins that are linked to it and holding it within the cuticle, may be a late target(s) for the CPs.

### Effects of CPs on *C. elegans* or *H. bakeri* visualised using scanning electron microscopy (SEM)

**Fig. 3:**
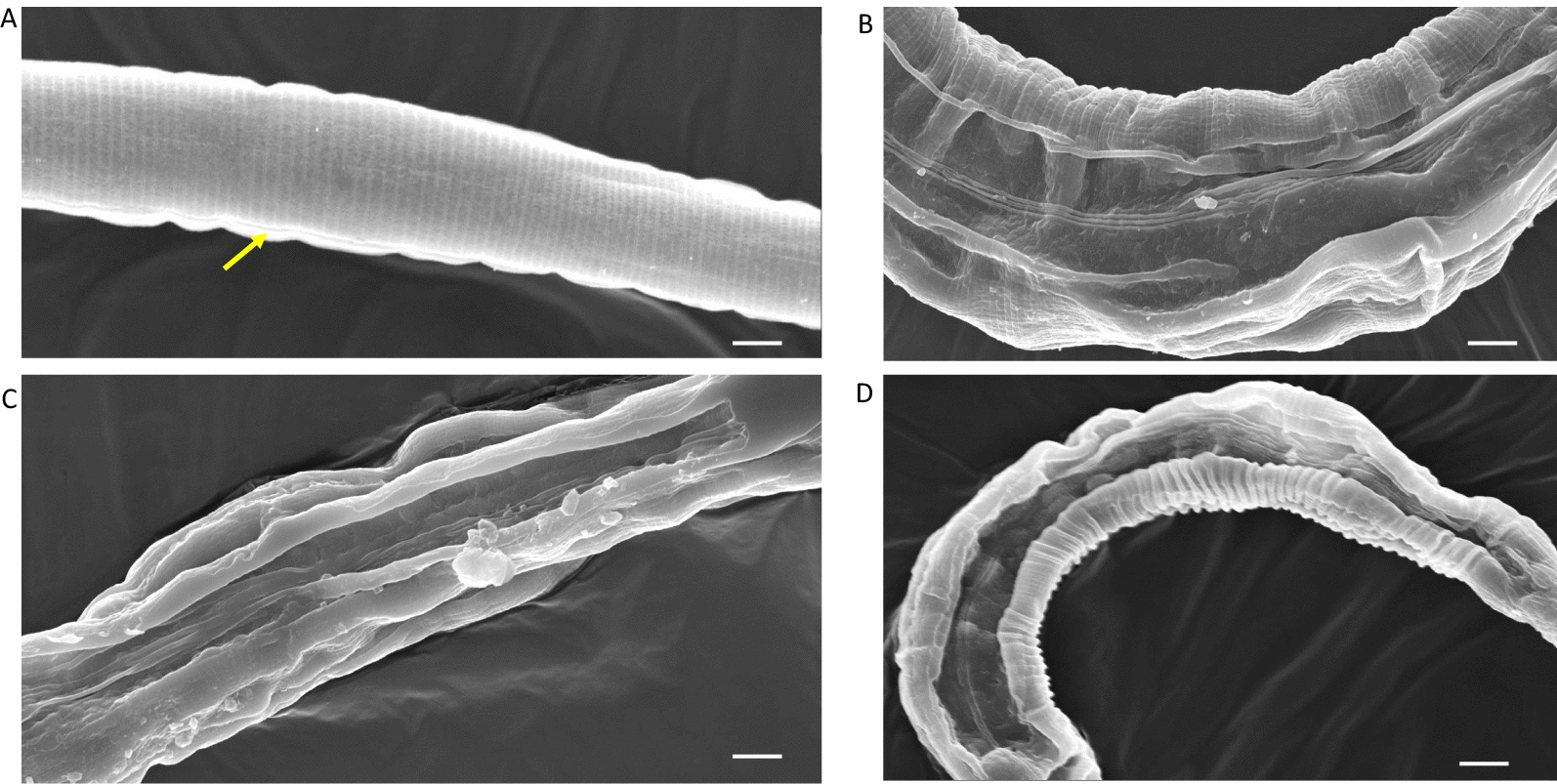
SEM of WT (Bristol N2) *C. elegans* after incubation in 1 µM papain or papain + E64 at time points of 10, 15 and 30 min. **a** The worms were apparently intact and unaffected when incubated with papain + E64 for 30 min, with the alae clearly visible (arrowed yellow). **b** After 10 min of incubation in papain, the cuticle appeared to be split longitudinally, which became more extensive at 15 min of incubation (**c**). By 30 min, the cuticle was split longitudinally, with a section of the cuticle totally destroyed or curled in on itself (**d**). Bar = 25 µm

In order to throw more light on the means by which CPs cause disruption of nematode cuticles, we went on to investigate by SEM the changes that occurred to adult *H. bakeri* cuticles, as well as *C. elegans* cuticles, including those of a knockout of a CP inhibitor (*cpi-2*) [30]. Whole WT or mutant *cpi-2(ok1256)* strains of *C. elegans* or *H. bakeri* were incubated with CP or CP + E64 at time points of 10, 15 and 30 min, then fixed and prepared for SEM. Figure 3 consists of electron micrographs of WT *C. elegans* incubated with 1 µM papain with or without a molar excess of E64. The WT *C. elegans* incubated with papain + E64 appeared to be intact (Fig. 3a), with the alae of the worm (arrowed yellow) running longitudinally along the worm’s body. This is in contrast with the worms incubated in 1 µM papain (b–d) where the cuticles have varying degrees of damage. At 10 min of incubation in papain, the cuticle surfaces of the WT worms were wrinkled and disrupted (Fig. 3b). The disruption was apparently extensive at 15 min of incubation in papain (Fig. 3c), whereas at 30 min, it appears that the worm has been split open longitudinally (Fig. 3d).

**Fig. 4:**
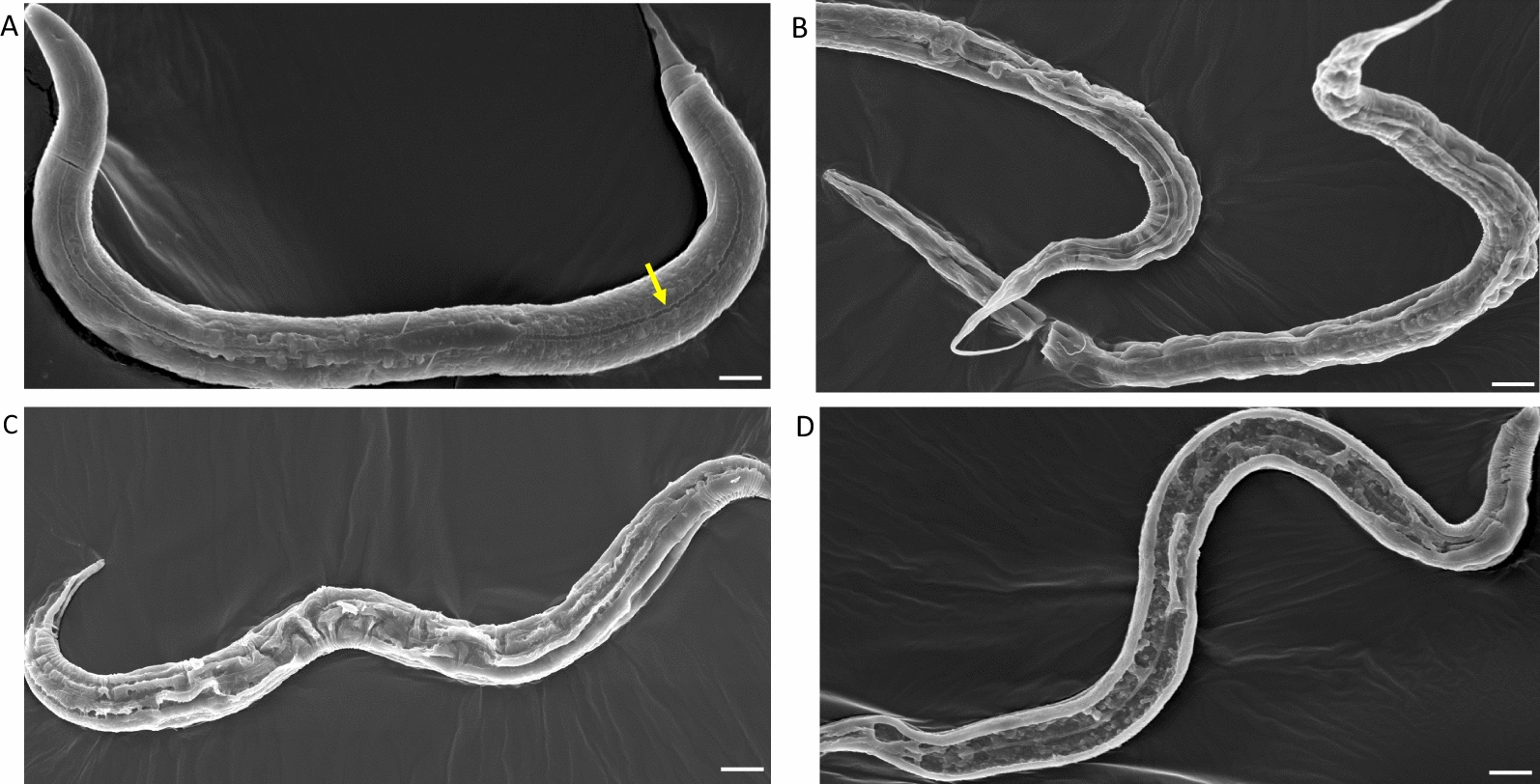
SEM images of cpi-2(RB1207, *ok1256*) C*. elegans* incubated in papain or papain + E64. **a** *cpi*-2 (RB1207, *ok1256*) *C. elegans* worms incubated in papain + E64 for 30 min retained their intact status with the alae (arrowed yellow) visibly running longitudinally along the worm. **b** At 10 min of incubation in papain, *cpi*-2(RB1207, *ok1256*) *C. elegans* showed wrinkling of the cuticles. **c** By 15 min, extensive blistering of the cuticles was apparent. At 30 min, most of the cuticle was lost or split along the alae, exposing the internal cavity (**d**). Bar = 50 µm

**Fig. 5:**
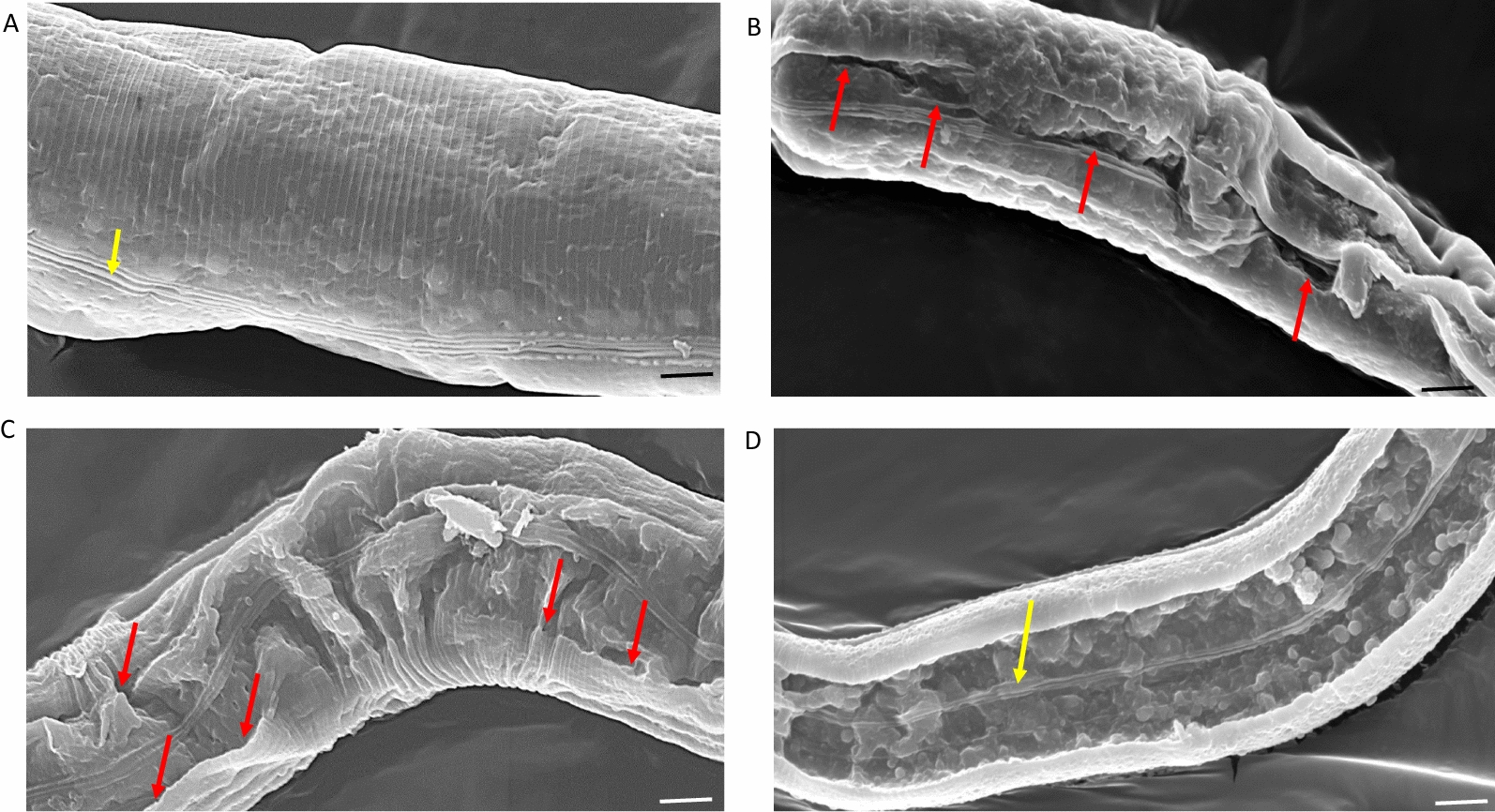
SEM images of *cpi*-2(RB1207, *ok1256*) *C. elegans* incubated in papain or papain + E64. **a** Worms incubated for 30 min in papain + E64 clearly showed the delicate intact structure of the cuticle, with the alae being clearly visible (yellow arrow). **b** By contrast, after 10 min of incubation, worms incubated in papain showed tearing or were split longitudinally along a line very close to the alae (arrowed red). **c** At 15 min, the papain-induced cuticle disruption probably caused the cuticles to detach from the rest of the body in what appeared to be sheets of cuticle (arrowed red). **d** After 30 min of incubation, the cuticle was often split longitudinally, in some cases, with the alae on the opposite side of the worm still intact (yellow arrow). Bar = 10 µm

The electron micrographs of *cpi-2(ok1256)* mutant *C. elegans* incubated in 1 µM papain or papain + E64 are shown in Figs. 4 and 5. Figure 4 illustrates the entire worms at low power, with the alae arrowed yellow in Fig. 4a. Figure 4b illustrates a worm after 5 min in papain, with wrinkling of the cuticle. After 15 min, extensive wrinkling and blistering of the cuticle can be seen (Fig. 4c). At 30 min, the cuticle has been split along the alae, with the cuticle on either side either folded over on itself or missing completely (Fig. 4d). At higher magnification, the *cpi-2(ok1256)* mutant *C. elegans* were damaged by papain (Fig. 5b–d) when contrasted to worms incubated in papain + E64, where the cuticle, including the alae (yellow arrow), appears to be intact, even after 30 min of incubation (Fig. 5a). At 10 min of incubation, worms incubated in papain showed tears longitudinally along the alae (arrowed red, Fig. 5b). The papain-induced tearing may have caused the cuticles to detach from the rest of the body by 15 min in what appear to be sheets of cuticle (arrowed red in Fig. 5c). A ribbon-like structure (arrowed yellow) appears to be the alae still intact on the opposite side of the worm, with the entire inner contents of the nematode having been lost by 30 min of incubation with papain (Fig. 5d).

**Fig. 6:**
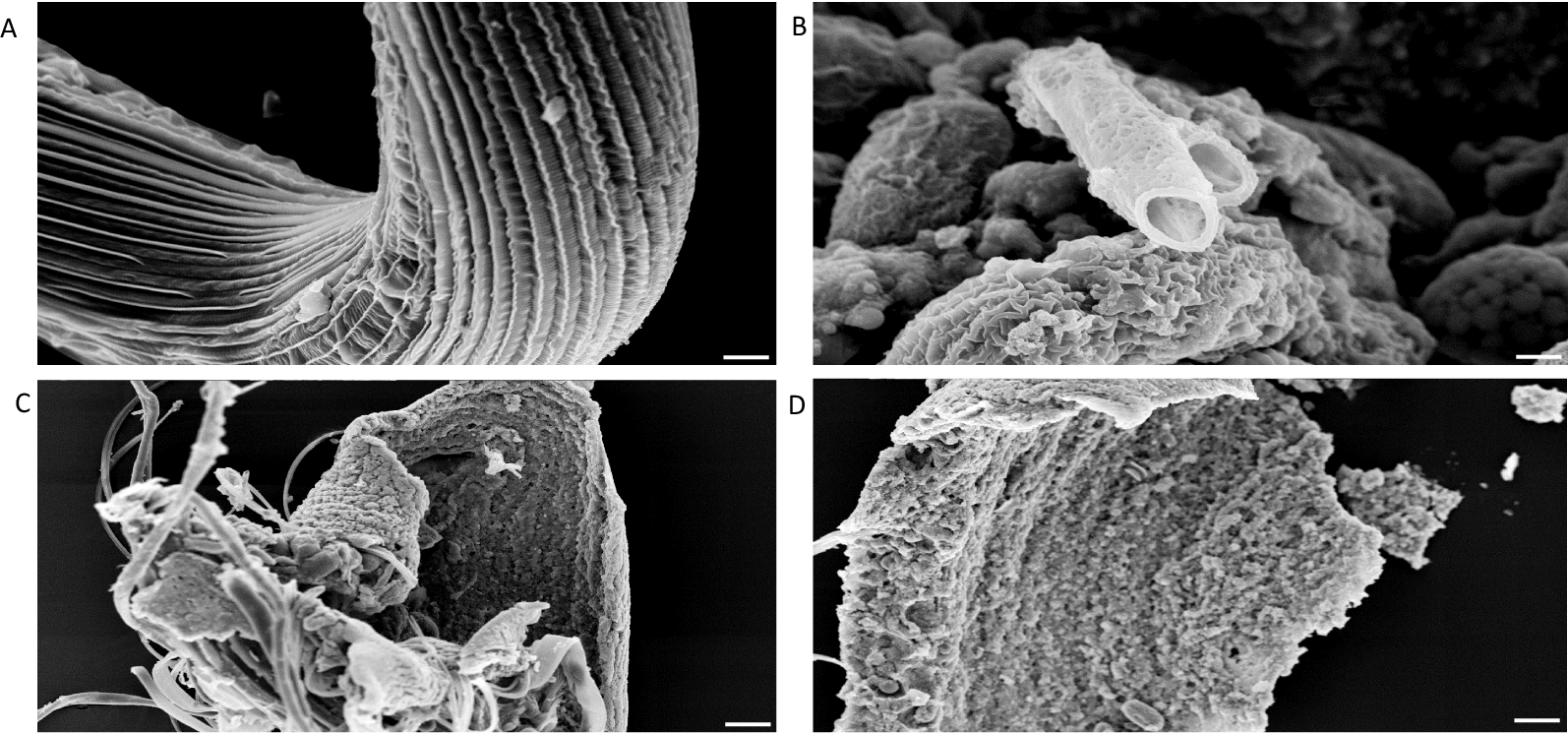
SEM of *H. bakeri* incubated in papain or papain + E64. *H. bakeri* incubated in papain + E64 for 30 min were intact and appeared undamaged (**a**), whereas worms incubated in 1 µM papain for 10 min or longer showed rapid and extensive digestion and were no longer recognisable (**b** 10, **c** 15 min). After 30 min of incubation, all that remained were sheets of insoluble material (**d**). Bar = 25 µm

Compared to *C. elegans*, when incubated with 1 µM papain, *H. bakeri* showed greater susceptibility to damage by CP at all the incubation times (Fig. 6). At 10 min of incubation, the worms already appeared totally digested with only a fragment of the gut being anatomically discernible (Fig. 6b). The worms were totally digested at 15 and 30 min of incubation with only the insoluble precipitates left after incubation (Fig. 6c and d). The damage to *H. bakeri* was caused by CP action as worms incubated in papain + E64 were not affected but retained their intact status (Fig. 6a).

### Target proteins for CPs on intact *H. bakeri* or prepared *H. bakeri* cuticles, or whole *C. elegans*

Nematode cuticles are substrates for CPs, and some cleaved products of hydrolysis are likely to be soluble. We analysed soluble products from prepared cuticles or whole *H. bakeri* incubated in CP or CP + E64, with SDS-PAGE. Bands that were unique in the papain digests or in the papain + E64 incubations were selected for in-gel trypsin digestion in conjunction with mass spectrometry analysis to identify the corresponding proteins (Additional file 1: Tables S1 and S2). Cuticle globin *(gi/8569651*), required for respiration by the nematode [45, 46], was one of the proteins identified using papain (Additional file 1: Table S1). In addition, a single peptide identified the structural protein CUT-19. However, as only a single peptide was identified, further validation is required. Cuticlins are major and important structural components of the nematode cuticle, and their hydrolysis is expected to lead to loss of integrity of the entire cuticle structure, weakening it sufficiently to enable its rupture through the high hydrostatic pressure within the pseudoceolomic cavity.

*Caenorhabditis elegans* or washed *C. elegans* cuticles were incubated with papain or papain + E64 (Additional file 1: Tables S3 and S4). Similarly, whole *C. elegans* or washed *C. elegans* cuticles were also incubated with PLS and PLS plus E64 (Additional file 1: Table S5). Following SDS-PAGE, the unique bands were selected for in-gel trypsin digestion in conjunction with mass spectrometry analysis to identify the corresponding proteins. Of particular interest was the identification of an important cuticle structural protein, COL-87. However, as the protein was identified by a single peptide, further validation is required.

## Discussion

In view of the threat of nematode resistance, our attention is on the development of drugs with multiple modes of action, i.e. with more than one target molecule and reduced likelihood of development of resistance. Focus has been on CPs and earlier reports of their effectiveness as anthelmintics [26–28, 47]. Although CPs attack and destroy nematode cuticles, the molecular target(s) and possible sites of activity on the structural proteins that constitute the cuticle have not been investigated.

For an anthelmintic based on CPs to be used on a large scale at an economic price, it is likely that a preparation such as PLS will be employed. This is a mixture of four closely related CPs, one of which is papain [36]. In order to simplify the interpretation of our data, particularly those using the proteomic approach, we decided to employ purified papain in our experiments alongside PLS. The effect of papain on the nematode cuticle is superficially similar to that of PLS, but it is unlikely that the two preparations will have identical effects.

DPY-7 collagen is a target for CPs on the cuticle of *C. elegans*. Our study found that the loss of DPY-7 immunoreactivity in *C. elegans* is time-dependent and that hydrolysis of DPY-7 or its disappearance by other means probably began before 5 min at a 1 µM concentration of CPs, whereas total loss of DPY-7 immunoreactivity appeared to take place when the worms were incubated longer in papain for up to 30 min. The time course of the loss of DPY-7 immunoreactivity on *C. elegans* was slow enough to allow us to use it to visualise anatomical disruption of the entire nematode cuticle by CP. The nematode cuticle is a multi-layered structure with about 80% of its protein as collagen [40]. DPY-7, DPY-2, DPY-3, DPY-8 and DPY-10 are obligate partners and are necessary in the formation of the thin thread-like structures needed for the genesis and maintenance of the annular furrows of *C. elegans* cuticles [39]. Therefore, loss of DPY-7 immunoreactivity by CP would suggest the destruction of the framework of the cuticle leading to loss of cuticular structure. This could be due to the hydrolysis by CP of any one or more of these components, or of others that have not yet been identified, resulting in collapse of the whole architecture of the cuticle, possibly seen as wrinkling on the surface as is usually associated with CP attack on the cuticles of parasitic nematodes [28]. As the time of incubation was increased, more of the DPY-7 and probably the other cuticle collagen proteins were hydrolysed, making the cuticle weaker, the physical result of which is the loss of integrity of the cuticle as seen with SEM and loss of DPY-7 immunoreactivity observed in immunohistochemical imaging of *C. elegans* incubated in CP. The disruption and digestion of the *H. bakeri* or *C. elegans* cuticles by CPs was a time-dependent but quite rapid process, producing severe damage to the cuticles. This suggests that there are many targets for CPs on nematode cuticles, most of which remained unidentified.

SEM demonstrated that dead *H. bakeri* are more susceptible to CP attack than dead *C. elegans*. An earlier report [30] indicated that the dose of CP that kills a parasitic nematode was unable to cause the death of wild type *C. elegans*. *C. elegans* possess CP inhibitors, presumably to protect against exogenous CPs in their external environments containing bacteria, fungi and decaying plant material [30]. With *H. bakeri*, Stepek et al. [48] observed cuticular damage after 15 min of incubating living *H. bakeri* in 200 μM papain, a 100-fold higher concentration than was used in this study. The difference in the amount of CP needed to cause cuticular damage to living and dead *H. bakeri* might be related to the presence or absence of cystatin secretions. *H. bakeri* cystatin(s) is involved in immunoregulation [17] and is presumed to be a secreted protein, so could influence CP activity if the animal is alive. The influence of cystatins in dead worms might be lessened by the inability to release cystatins from a store elsewhere in the worm and mobilised to the cuticles as may occur in the living nematode, as seems to be the case in live *C. elegans* [30]. As components of secretory products of parasitic nematodes, cystatins may be deposited in the cuticles [17, 48, 49]. In the situation where there are cystatins within the cuticles, our cuticle preparation would most likely have removed any cystatins, making the cuticles more susceptible to the action of CPs.

A cuticle-related protein, extracellular cuticle globin, was identified from *H. bakeri* samples incubated with CPs. Its absence in the cuticles incubated in papain + E64 indicates that it was released by papain. This extracellular cuticle globin has high-affinity oxygen binding and is required by the parasitic nematodes to obtain oxygen in their near anaerobic environment within the host gut [50]. Disruption of cuticle globin by papain would disengage the mechanism through which the worm obtains oxygen from its host. We therefore conclude that CPs are able to disrupt the mechanism of oxygen uptake from the host, another potential killing method.

Nematode cuticle structural proteins are held together by covalent tyrosine cross-links [51]. The failure to identify many structural proteins is likely to be due to the inability of MS software to identify peptides containing tyrosine cross-links. The presence of the cross-links as well as the likelihood that many of the cross-links are formed between different cuticular collagen and cuticlin gene products will make the resulting structure impossible for the software to recognise. The only peptides that could be recognised would be those that do not contain tyrosine cross-links and are the product of a single gene. In *C. elegans*, about 160 and at least 8 functionally defined genes encode for cuticle collagens and cuticlins, respectively [40, 52], which are all likely to be substrates for the formation of tyrosine-based cross-links [15, 53].

We identified a single peptide from each of two cuticle structural proteins, COL-87 and CUT-19, in digests of *H. bakeri* and *C. elegans*, which may suggest that, along with DPY-7, these structural proteins may be cuticular targets for CPs. No examples of structural cuticle components were found in any of our control samples where the action of the CPs was blocked by the irreversible CP inactivator E64. Other proteomic analyses of nematodes have failed to identify significant numbers of peptides from cuticle proteins [42, 46], presumably for the reasons outlined above. For these reasons, we consider COL-87 and CUT-19, along with DPY-7, to be possible CP targets in the cuticle.

The pattern of activity of CPs on nematodes is evidently novel and involves the targeting of a number of different gene products, making resistance of nematodes to anthelmintics derived from CPs difficult to achieve. We therefore suggest that CPs are good candidates for an anthelmintic with a completely novel mode of action from those attributed to other anthelmintics, and that development of resistance against CPs by nematodes will be slow as it will probably require simultaneous mutations of a number of different genes encoding collagens, cuticlins, and possibly other essential components of the nematode cuticle.

## Supplementary Information

**Additional file 1.** Contains additional methodology and tabulated results.

## Abbreviations

CP: Cysteine proteinase
DTT: Dithiothreitol
E64: L-*trans*-epoxysuccinyl-leucylamido(4-guanidino)butane
HBSS: Hank’s balanced salt solution
LC/MS/MS: Liquid chromatography-tandem mass spectrometry
PBS: Phosphate-buffered isotonic saline
PLS: Papaya latex supernatant
SEM: Scanning electron microscopy
SDS-PAGE: Sodium dodecyl sulphate–polyacrylamide gel electrophoresis
TBST: Tris-buffered saline with Tween-20
WT: Wild type
